# Involvement of Bradykinin Receptor 2 in Nerve Growth Factor Neuroprotective Activity

**DOI:** 10.3390/cells9122651

**Published:** 2020-12-10

**Authors:** Carla Petrella, Maria Teresa Ciotti, Robert Nisticò, Sonia Piccinin, Pietro Calissano, Simona Capsoni, Delio Mercanti, Sebastiano Cavallaro, Roberta Possenti, Cinzia Severini

**Affiliations:** 1 Institute of Biochemistry and Cell Biology, National Research Council, Sapienza University of Rome, Viale del Policlinico, 155-00161 Rome, Italy; carla.petrella@cnr.it (C.P.); mariateresa.ciotti@cnr.it (M.T.C.); delio.mercanti@cnr.it (D.M.); 2 Department of Biology, University of Rome “Tor Vergata”, Via della Ricerca Scientifica 1, 00133 Rome, Italy; robert.nistico@gmail.com (R.N.); sonia.piccinin@gmail.com (S.P.); 3 Rita Levi-Montalcini European Brain Research Institute (EBRI), Viale Regina Elena, 295, 00161 Rome, Italy; pietro.calissano@gmail.com; 4 Section of Physiology, Department of Biomedical and Specialty Surgical Sciences, University of Ferrara, Via Luigi Borsari 46, 44121 Ferrara, Italy; simona.capsoni@unife.it; 5 Bio@SNS Laboratory of Biology, Scuola Normale Superiore, Piazza dei Cavalieri, 7, 56126 Pisa, Italy; 6 Institute for Biomedical Research and Innovation, National Research Council, Via Paolo Gaifami 18, 95126 Catania, Italy; sebastiano.cavallaro@cnr.it; 7 Department Medicine of Systems, University of Rome “Tor Vergata”, Via della Ricerca Scientifica 1, 00133 Rome, Italy; roberta.possenti@gmail.com

**Keywords:** nerve growth factor (NGF), bradykinin receptor 2 (B2R), microglial cells, LTP, Alzheimer’s disease animal models

## Abstract

Neurotrophin nerve growth factor (NGF) has been demonstrated to upregulate the gene expression of bradykinin receptor 2 (B2R) on sensory neurons, thus facilitating nociceptive signals. The aim of the present study is to investigate the involvement of B2R in the NGF mechanism of action in nonsensory neurons in vitro by using rat mixed cortical primary cultures (CNs) and mouse hippocampal slices, and in vivo in Alzheimer’s disease (AD) transgenic mice (5xFAD) chronically treated with NGF. A significant NGF-mediated upregulation of B2R was demonstrated by microarray, Western blot, and immunofluorescence analysis in CNs, indicating microglial cells as the target of this modulation. The B2R involvement in the NGF mechanism of action was also demonstrated by using a selective B2R antagonist which was able to reverse the neuroprotective effect of NGF in CNs, as revealed by viability assay, and the NGF-induced long-term potentiation (LTP) in hippocampal slices. To confirm in vitro observations, B2R upregulation was observed in 5xFAD mouse brain following chronic intranasal NGF treatment. This study demonstrates for the first time that B2R is a key element in the neuroprotective activity and synaptic plasticity mediated by NGF in brain cells.

## 1. Introduction

Neurotrophin nerve growth factor (NGF) is characterized by the ability to improve the growth and differentiation of sensory and sympathetic nerve cells [1]. Numerous papers have proposed NGF as a possible therapeutic option in the treatment of Alzheimer’s disease (AD) due to its ability to sustain cholinergic activity [2,3,4] and its neuroprotective function [5,6], together with its ability to directly inhibit amyloidogenesis [7,8].

However, the clinical application of NGF in AD is limited by its poor bio-distribution to the brain after systemic delivery [9] and its potent pain sensitizing activity after systemic exposure [10,11].

Indeed, NGF plays an important role in inflammatory pain by driving peripheral sensitization, directly acting on the peripheral terminal to produce heat hyperalgesia [12,13,14].

As demonstrated in dorsal root ganglion sensory neurons (DRGs), long-term exposure to NGF profoundly regulates the gene expression of several functionally important proteins, including neurotransmitters, receptors, and voltage-regulated ion channels involved in pain transduction [11,15].

Among nociceptive-related peptides released by the activation of nociceptive neurons, bradykinin (BK) is the most potent endogenous pain-producing substance known [16,17,18].

The biological effects of BK are produced by the activation of two transmembrane receptors coupled to G proteins (Gα and Gq), namely B1 and B2 receptors (B1R and B2R) [19]. The majority of the activity of BK is mediated by B2R, which has high affinity for BK and is considered a constitutive receptor [20]. On the other hand, B1R possesses higher affinity for des-Arg9-BK and Lys-des-Arg9-BK, and has limited distribution in tissues under physiological conditions. However, it is highly expressed in pathological conditions, such as chronic inflammation, infection or injury [19].

In DRGs, constitutive B2R has been demonstrated to be strongly upregulated after NGF treatment, because both the number of B2R binding sites and the physiological response to BK increase, alongside the incubation time with NGF [21,22].

However, the involvement of BK receptors related to the neuroprotective activity of NGF has never been demonstrated in central, nonsensory neurons.

A considerable body of evidence has established the role of BK and its receptors in AD human pathology and animal models, as recently reviewed [23,24,25].

In cultured skin fibroblasts from AD patients, the overexpression of BK receptors has been established [26], together with increased activity of B2R; these modifications are able to improve the cellular response to BK which occurs during AD neuroinflammation [27].

In AD animal models, it was shown that chronic intracerebroventricular (icv) injection of Aβ in rats provoked a significant enhancement of both B1R and B2R binding sites, mainly in brain regions associated with cognitive behavior [28]. Moreover, after a single icv injection of aggregated Aβ, a selective increment of B1R, without variation of B2R expression, was observed in the hippocampus and prefrontal cortex of mice [29].

The increased expression of B1R in reactive astrocytes surrounding Aβ plaques in the hippocampus of 10-month-old APP mice (J20 line), and the ability of B1R antagonism to reduce amyloidosis and cerebrovascular and memory deficits, serve as evidence for the harmful role of inducible B1R in AD neuroinflammation [30].

However, conflicting results about the role of B1R in AD pathogenesis have been reported. Indeed, it has been shown that intranasal treatment with a B1R antagonist enhances Aβ deposits and microglia/macrophages activation in two-month-old 5X familial AD mice [31] and in eight-month-old Tg-SwDI mice [32], indicating a protective role of B1R.

Since B1R is induced by neuroinflammation, these variable results suggest that the involvement of the B1R in AD pathogenesis could depend upon the AD animal model or the disease progression at the time of treatment. Overall, while inducible B1R is involved in the neuroinflammation related to AD, constitutive B2R seems to preferentially mediate its neuroprotective effects, as confirmed by experiments performed in BKRs knock-out mice treated with Aβ. Following chronic Aβ infusion, B2R knock-out (koB2) mice showed significant premature memory impairment compared to control animals (WT) with the same treatment, while B1R knock-out (koB1) mice did not show any difference in memory impairment caused by Aβ [32]. These results were confirmed evaluating not only the cognitive behavior, but also the number of Aβ deposits in koB1 and koB2 mice brain following chronic Aβ infusion. In koB2 mice, an increased number of Aβ plaques was found compared to WT and koB1 treated mice, pointing to B2R as a potential therapeutic target in AD [33].

The aim of the present work is to investigate the involvement of B2R in the mechanism of action of NGF in vitro in cortical primary cultures and in hippocampal slices, and in vivo in AD transgenic mice chronically treated with NGF.

## 2. Materials and Methods

### 2.1. Chemicals

Recombinant human NGF was obtained from Xiamen Bioway Biotech Group Co., (Xiamen, Fujian, China). Cell culture media were obtained from Invitrogen (Milano, Italy). All other reagents were from Sigma (St. Louis, MO, USA) if not stated otherwise.

### 2.2. Mixed Cortical Cultures (CNs), Treatment and Viability Assay

All procedures were approved by the Italian Ministry of Health (Rome, Italy) and performed in compliance with the guidelines of the US National Institutes of Health and the Italian Ministry of Health (D.L.116/92, approved on 28 June 2017). Mixed cortical cultures containing both neurons and glial cells (astrocytes and microglia) were prepared from brains of embryonic day 17–18 (E17/E18) embryos from timed pregnant Wistar rats (Charles River, Wilmington, MA, USA), as previously reported [34]. In brief, cortex was dissected in Hanks’ balanced salt solution buffered with Hepes and dissociated via trypsin treatment. Cells were plated at 1 × 10^6^ cells on 3.5-cm dishes precoated with poly-L-lysine. After two days of culturing in Neurobasal medium with 1% B27 supplement (0.5 mM L-glutamine, 1% antibiotic penicillin/streptomycin), half of the medium was changed every 3–4 days. All experimental treatments (NGF 100 ng/mL) were performed on 10-day “in vitro” (DIV) cultures in Neurobasal + 1% B27 fresh medium for 48 h. To obtain NGF deprived cells, CNs treated with NGF were incubated with Anti-Nerve Growth Factor [alphaD11] (absolute Antibody, UK: Ab00278-6.1) at a 30-fold higher concentration with respect to NGF. The culture cell composition was determined using immunocytochemical staining for neurons (NeuN antibody, (Sigma, St. Louis, MO, USA 1:200), astrocytes (GFAP antibody, 1:400, Sigma) and microglia (Iba1 antibody, Abcam Cambridge, UK, 1:200) with 4′,6-diamidino-2-phenylindole (DAPI) nuclear staining (Thermo Fisher Scientific, Waltham, MA, USA). Mixed cultures contain about 45% NeuN+ cells, 50% GFAP+ cells, and 4% of Iba1+ cells.

Neuronal viability was assessed by counting the number of intact nuclei, as previously described [35,36]. Culture medium was removed and replaced by 0.5 mL of a detergent containing lysing solution (0.5% ethylhexadecyldimethylammonium bromide, 0.28% acetic acid, 0.5% Triton X-100, 3 mM NaCl, 2 mM MgCl_2_, in PBS pH 7.4 diluted 1/10). After a few minutes, the cells were collected, and intact viable nuclei were counted using a hemocytometer, since the detergent-containing solution is able to dissolve the nuclei of the cells that are dying, while healthy cells appear as phase-bright intact circles surrounded by a dark ring. Broken or damaged nuclei were not included in the counts.

### 2.3. Enriched Microglial Cultures

Mixed neural cell cultures, containing both neurons and glial cells (astrocytes and microglia) were prepared as mentioned above from rat cortex. Cells were plated at 3 × 10^6^ cells on 9-cm dishes without poly-L-lysine in DMEM (Gibco, Dublin, Ireland) containig glutamax (Gibco) and 10% fetal bovine serum (FBS) (Gibco). After five days of culturing, the medium was changed to eliminate dead neurons.

After 12 days of “in vitro” (DIV) culturing, floating microglial cells were removed by shaking the plate and used to obtain enriched microglia cultures. Microglia cells were plated at 2.5 × 10^5^ cells on 3.5-cm dishes precoated with poly-L-lysine in DMEM containing glutamax and 10% of FBS. After 48 h, the medium was changed and replaced with DMEM containing glutamax, 1% antibiotic penicillin/streptomycin and 0.1% of FBS. NGF was added at a dose of 100 ng/mL for a further 48 h.

### 2.4. Microarray Analysis

After ten DIV, cortical cultures were treated with NGF (100 ng/mL) for 72 h (+NGF).

Following this period, CNs were washed twice with Neurobasal + 1% B27 medium and incubated for 6 h (−NGF 6 h), while other cultures were treated with NGF (100 ng/mL) for the same period (+NGF 6 h).

Total RNA was extracted with Trizol (Invitrogen, Milano, Italy) from four biological replicates (derived from the same litter) for each of the experimental conditions (CTR, +NGF, −NGF 6 h and +NGF 6 h).

RNA integrity was confirmed using a RNA chip and a 2100 Bioanalyzer (Agilent Technologies, Santa Clara, CA, USA) with the protocol outlined by the manufacturer. Complementary RNAs (cRNAs) labeled with Cy3-CTP were synthesized from 1 μg of total RNA of each sample using the Low RNA Input Fluorescent Linear Amplification Kit (Agilent Technologies), following the manufacturer’s protocol. Aliquots (750 ng) of Cy3 labeled cRNA targets were hybridized on Whole Rat Genome Oligo Microarrays (Agilent Technologies). Microarray hybridization and washing were performed using reagents and instruments (hybridization chambers and rotating oven), as indicated by the manufacturer. Microarrays were scanned at 5-μm resolution using a GenePix Personal 4100A microarray scanner and the GenePix Pro 6.0 acquisition and data-extraction software (Molecular Devices, San Jose, CA, USA). Raw data were processed and analyzed by GeneSpring GX 11.5 software 13 (Agilent Technologies).

Gene symbol: *Kng1*, *Bdkrb2* and *Bdkrb1*.

Probe set id: *Kng1* (A_44_P348781), *Bdkrb2* (A_64_P036715) and *Bdkrb1* (A_64_P118628).

mRNA: *Kng1* (NM_012696), *Bdkrb2* (NM_001270713) and *Bdkrb1* (NM_030851).

### 2.5. Immunocytochemistry

Cultured cells were washed in PBS and fixed in 4% (*w/v* in PBS) paraformaldehyde for 30 min at room temperature. Fixed cells were washed in PBS, pH 7.4, permeabilized using 0.1% Triton X100-Tris-HCl, pH 7.4, for 10 min, and then treated with the following primary antibody: rabbit antiB2R (Alomone labs, Jerusalem BioPark, Israel, 1:200), mouse anti-MAP2 (Cell Signaling, Danvers, MA, USA 1:200), mouse anti-GFAP (Sigma Aldrich, St. Louis, MO, USA 1:400) or Goat-anti-IBA1 (Cell Signaling 1:200). After an overnight incubation at 4 °C, cells were washed in PBS and incubated with TRITC or FITC conjugated secondary antibody (Sigma Aldrich, 1:1000) for 30 min at room temperature. Nuclei were stained with Hoechst 33258 (0.25 μg/mL) for 5 min at room temperature. Controls to assess primary antibody specificity were performed by including the omission of the primary antibody.

Cells were visualized by conventional epifluorescence microscope (Olympus BX51; Milano, Italy) (40× objective).

### 2.6. ELISA

For the determination of bradykinin (BK) levels, cell media were loaded directly onto enzyme-linked immunosorbent assay (ELISA) BK plates, in accordance with the manufacturer’s instructions (Enzo Life Sciences, Ann Arbor, MI, USA). BK concentration was corrected by referring to the volume of the collected sample (1 mL). The minimum detectable level for this assay was 10 pg/mL. This assay can recognize B2 receptor ligands such as BK and Lys-BK, whereas it does not recognize B1 receptor ligands (i.e., [desArg9]-BK and [desArg9]-Lys-BK).

### 2.7. Electrophysiology

Mice (three-months old) were anesthetized with halothane and their brains were removed and placed in ice-cold artificial cerebrospinal fluid solution (ACSF) containing 124 mM NaCl, 2.5 mM KCl, 1.3 mM MgSO_4_, 1.25 mM NaH_2_PO_4_, 26 mM NaHCO_3_, 2.4 mM CaCl_2_, and 10 mM glucose. Hippocampal slices (350 μm) were cut with a Vibroslice (VT 1000S; Leica, Wetzlar, Germany) and kept for 1 h in oxygenated medium at room temperature (20–22 °C) before recordings. A single slice was then placed on a nylon mesh, completely submerged in a small chamber (0.5 mL), and superfused with oxygenated ACSF (30 °C) at 3 mL/min constant flow rate. Experiments were performed in the CA1 region and field excitatory synaptic potentials (fEPSPs) were recorded in stratum radiatum by stimulating Schaffer collaterals. Long-term potentiation (LTP) was induced by conventional HFS applied to the Schaffer collateral-CA1 synapses (1 train of 100 Hz). All data are presented as mean ± SEM and assessed for significance using the unpaired Student’s t test.

### 2.8. Transgenic Mice

Transgenic mice (three months old) with five familial Alzheimer’s disease mutations (5xFAD) and coexpressing FAD mutant forms of human APP and presenilin 1 were purchased from the Jackson Laboratory [37].

The 5xFAD mice used were hemizygotes with respect to the transgenes, while nontransgenic wild-type littermates were used as controls. Genotyping was performed by a PCR analysis of tail DNA. All analyses were done blind with respect to the genotype of the mice and treatment. All experiments with mice were performed according to the national and international laws for laboratory animal welfare and experimentation (EU directive no. 2010/63/EU and Italian DL no. 26 04/03/2014). Mice were kept under a 12 h dark to light cycle, with food and water ad libitum.

### 2.9. Intranasal Treatment with NGF and Tissue Processing

NGF diluted in 1M PBS was administered intranasally to mice, 3 µL at a time, alternating nostrils, with a lapse of 2 min between each administration, for a total of 14 times at a dose of µg/kg (equivalent to 0.51 pmol), as previously described by Capsoni et al. [38].

As control treatments, wild-type and 5xFAD mice were treated with PBS. The frequency of administration for intranasal delivery was three times per week (every two days). Administrations were repeated nine times over a 21-day period, followed by seven days of washout during which mice were not dosed with the protein. Following anesthesia with 2,2,2-tribromethanol, the caudal part of the brain was removed, stored at −80 °C, and used for Western blot analysis.

### 2.10. Western Blotting

For cytoplasmic lysates, cells were washed twice with ice-cold PBS, lysed in lysis buffer (1% NP40, 50 mM Tris-HCl, pH 8) containing 1X protease inhibitor mixture, and centrifuged at 10,000 rpm for 10 min at 4 °C. Samples were stored at −80 °C until analysis. To obtain a membrane-enriched fraction, brains were first mechanically homogenized in 250 μL of 50-mm Tris-HCl, pH 7.6, 0.01% Igepal, 150-mm NaCl, 2-mm EDTA, 0.1% SDS, and 1× protease inhibitor cocktail (Roche, Basilea, Switzerland) using five passes through a 1 mL syringe with an 18 gauge needle. After centrifugation, the remaining pellet was then mechanically dissociated by trituration with a Gilson micropipette in 250 μL of TNT buffer (50 mm Tris-HCl, pH 7.4, 150 mm NaCl, 0.1% Triton X-100, and 1× protease inhibitor cocktail). The supernatant of this extract was obtained following 90 min at 16,100× *g*, 4 °C. The remaining pellet was then triturated with a micropipette followed by passive lysis on a rotating platform for 15 min at 4 °C in 250 μL of RIPA buffer (50 mm Tris-HCl, pH 7.4, 150 mm NaCl, 0.5% Triton X-100, 1 mm EDTA, 3% SDS, 1% sodium deoxycholate, and 1× cocktail inhibitor). Membrane-enriched proteins were collected from the supernatant of this extract following two centrifugations for 90 min each at 16,100× *g*, 4 °C.

Protein concentration was measured using a Biorad DC protein assay kit (Bio-Rad, Hercules, CA, USA), and equivalent amounts of protein (30 μg) were separated on 4–12% Bis-Tris NuPage precast gels (Invitrogen), blocked with 5% milk in TBS-Tween for 1 h and then incubated overnight with rabbit anti-B2R antibody (Alomone labs 1:1000) or mouse anti-β-actin (Sigma Aldrich 1:10,000). Incubation with goat anti-rabbit or rabbit anti-mouse secondary antibodies peroxidase-coupled was performed for 1 h at room temperature. Immunoreactivity was developed with enhanced chemiluminescence (ECL system; Amersham, Arlington Heights, IL, USA). Protein loading was monitored by normalization to β-actin. Blots were scanned, and a quantitative densitometric analysis was performed using ImageJ software (http://imagej.nih.gov/ij/).

### 2.11. Data Analysis

A statistical analysis was performed using SPSS 11.0.0 for Windows (SPSS Inc., Chicago, IL, USA). All results are expressed as mean ± SEM, with *n* the number of independent experiments. The significance of the effect was performed by one-way analysis of variance (ANOVA) followed by Bonferroni’s test for multiple comparison. The significance level was set at *p* < 0.05 (*) and *p* < 0.01 (**).

## 3. Results

### 3.1. Expression of Bradykinin (BK) and BK Receptors in CNs Following NGF Treatment and Deprivation

**Figure 1:**
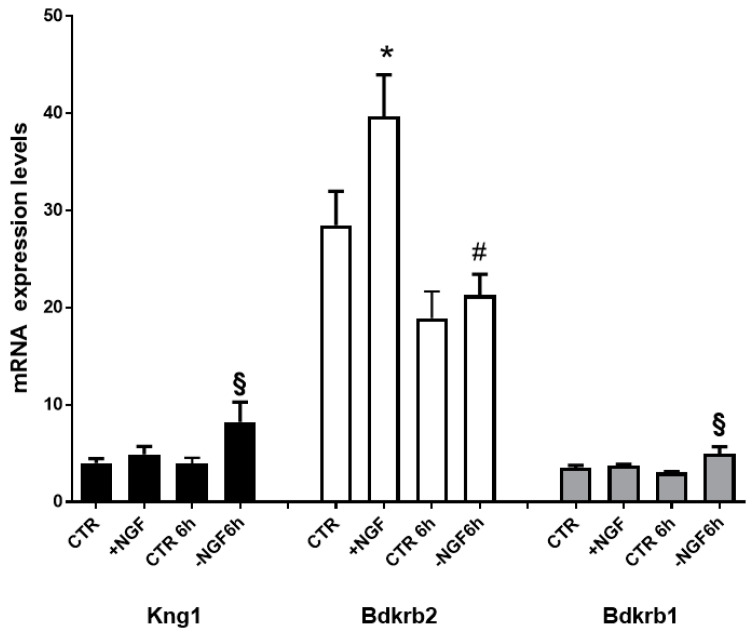
mRNA expression profiles of BK and its receptors genes after NGF treatment, deprivation, and rescue. Transcript levels of genes encoding BK (*Kng1*; NM_012696), Bradykinin receptor 2 (*Bdkrb2*; NM_001270713), and Bradykinin receptor 1 (*Bdkrb1*; NM_030851) in cortical neurons (CNs) following 48 h NGF treatment (+NGF), induction of apoptosis by 6 h NGF deprivation (−NGF 6 h), and rescue by 6 h NGF replacement (+NGF 6 h). Data represent means (±S.E.M.) from four replicates. Statistically significant differences were calculated by one-way analysis of variance (ANOVA) followed by Bonferroni’s test for multiple comparison (* *p* < 0.001 vs CTR; § *p* < 0.05 vs CTR 6 h; # *p* < 0.05 vs. +NGF replacement (+NGF).

Aiming to define the transcriptional changes regulating BK and its receptor proteins involved in NGF treatment, deprivation, and rescue, we measured the steady-state mRNA levels of gene encoding for BK (Kng1), B1R (Bdkrb1), and B2R (Bdkrb2). As shown in Figure 1, Bdkrb2 was the only upregulated gene following NGF treatment, while following NGF deprivation, we observed significantly increased levels of both Kng1 and Bdkrb1.

Together, these data suggest that NGF regulates BK and its receptor system, specifically acting through the constitutive B2R in neuroprotective conditions and through BK and the inducible B1R in apoptosis due to NGF deprivation.

### 3.2. Steady-State Levels of B2R Protein

**Figure 2:**
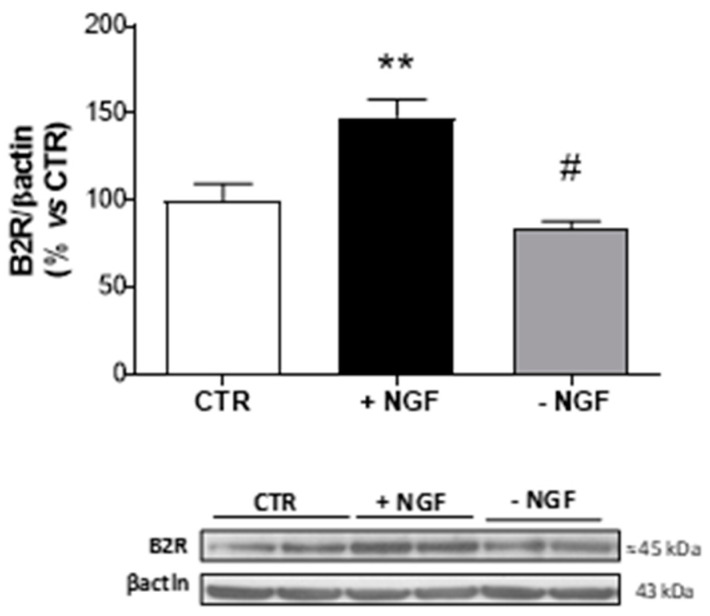
Expression of B2R after NGF treatment and deprivation in CNs. At 10 DIV, CNs cultured in Neurobasal + 1% B27 (CTR) were treated for 48 h with NGF (100 ng/mL, +NGF) before being deprived of NGF by anti-NGF antibody treatment and incubated for 24 h (−NGF). B2R protein expression was measured by Western blot analysis. The immunoreactive signals at 45 kDa were quantified and normalized against β-actin and expressed as a percentage of the control (CTR). Data represent means (±S.E.M.) from four independent experiments run in duplicate. Statistically significant differences were calculated by one-way analysis of variance (ANOVA) followed by Bonferroni’s test for multiple comparisons (** *p* < 0.01 versus CTR; # *p* < 0.01 versus NGF treatment).

Since *Bdkrb2* was the only increased gene following NGF treatment, we analyzed the corresponding B2R expression level in CNs lysates by Western blot analysis. As shown in Figure 2, NGF treatment provoked a significant increase (about 1.5 fold upregulation), while NGF deprivation caused a significant reduction in protein expression (about 3-fold downregulation) of B2R, thus confirming the microarray data.

**Figure 3:**
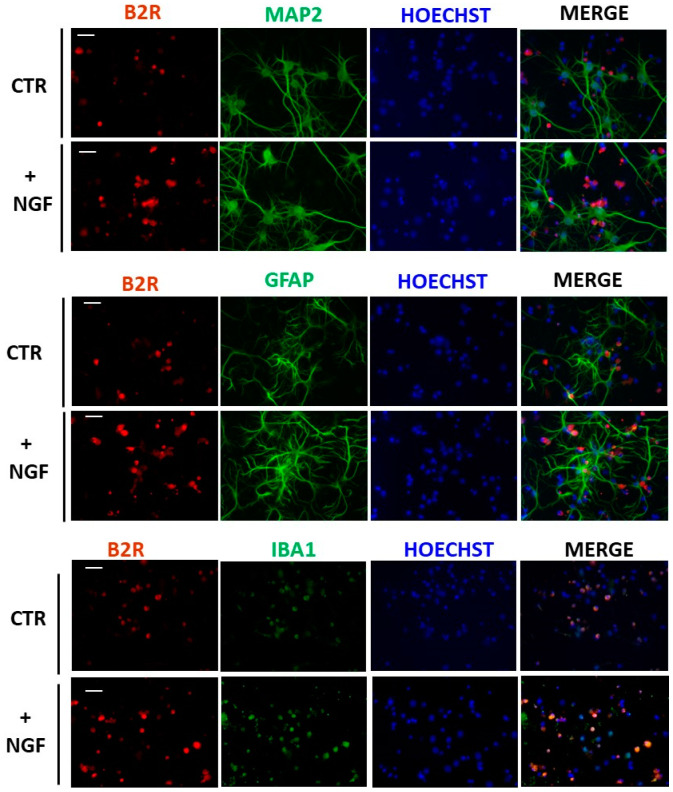
Expression of B2R in mixed cortical cultures. Representative immunofluorescence images of cultured CNs stained with antibodies for B2R (red), neurons (MAP2, green), astrocytes (GFAP, green), or microglial cells (Iba1, green) and nuclei (Hoechts, blue) in control conditions (CTR) and after NGF treatment (+NGF). B2R immunoreactivity significantly increased in microglial cell body after NGF treatment. Scale bar: 15 µm.

Furthermore, an immunofluorescence analysis of CNs revealed the exact localization of B2R in the basal condition after NGF treatment. As shown in Figure 3, a merged analysis indicated that B2R immunoreactivity was present in microglial cells (IBA1) in control conditions (CTR) and overexpressed after NGF treatment (+NGF). Conversely, no colocalization was detected in neurons (MAP2 cells) or astrocytes (GFAP cells).

### 3.3. Expression of B2R in Cultured Microglial Cells

**Figure 4:**
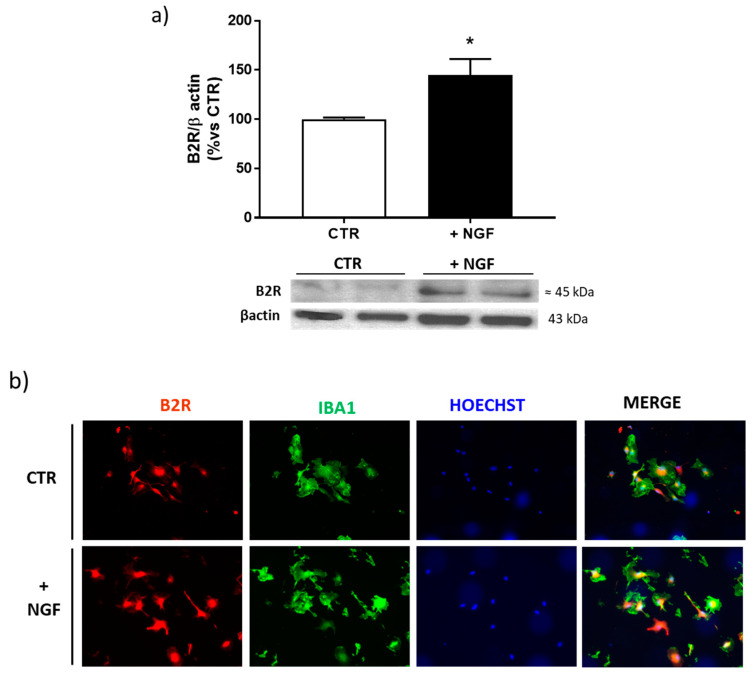
Expression of bradykinin receptor 2 (B2R) after NGF treatment in cortical microglial cells. (**a**) Western blot analysis of B2R protein expression in enriched microglial cells cultures treated for 48 h with NGF 100 ng/mL (+NGF), normalized against β-actin, and expressed as a percentage of the control (CTR). Data represent means (±S.E.M.) from four independent experiments run in duplicate. Statistically significant differences were calculated by one-way analysis of variance (ANOVA) followed by Bonferroni’s test for multiple comparisons (* *p* < 0.05). (**b**) Representative immunofluorescence images of microglial cells stained with antibodies for B2R (red) or microglia (Iba1, green) and nuclei (Hoechts, blue) in control conditions (CTR) and after NGF treatment (+NGF). Scale bar: 15 µm.

Since B2R seems to be selectively upregulated by NGF treatment in microglia, we performed Western blot and immunofluorescence analyses of this receptor in enriched microglial cultures. As shown in Figure 4a,b, B2R was significantly upregulated, confirming the results obtained in mixed cortical cultures.

### 3.4. CNs Viability

**Figure 5:**
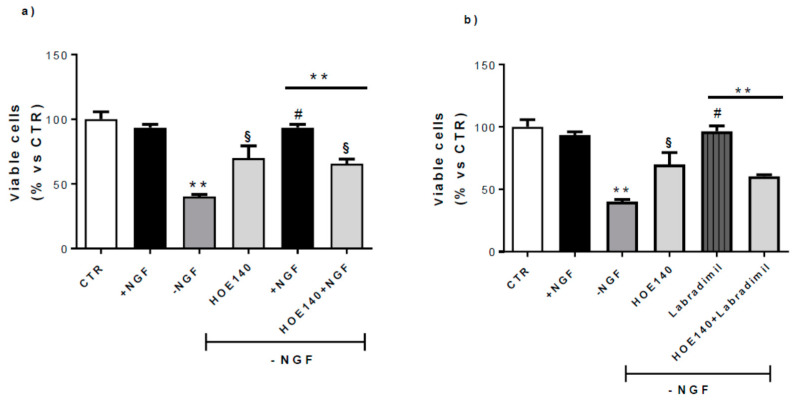
Effect of B2R antagonism on CN viability. At 10 DIV, CNs cultured in Neurobasal + 1% B27 (CTR) were treated for 48 h with NGF 100 ng/mL (+NGF) and afterwards deprived of NGF for 24 h by washing (−NGF) or rescued by 24 h NGF replacement (+NGF). Neuronal viability was determined by counting intact nuclei and expressed considering 100 as the number of viable neurons in CTR conditions. (**a**) Treatment with HOE140 (1 µM), a selective B2R antagonist, induced a considerable inhibitory effect on the neuroprotection promoted by NGF replacement (HOE140 +NGF). (**b**) In the same conditions, HOE140 significantly antagonized the neuroprotective activity exerted by RPM-7, a selective B2R agonist (RPM-7 (100 nM). Data represent means (±S.E.M.) from four duplicate experiments. Statistically significant differences were calculated by one-way analysis of variance (ANOVA) followed by Bonferroni’s test (** *p* < 0.01 versus +NGF; # *p* < 0.01 versus −NGF; **^§^** *p* < 0.01 versus −NGF).

Considering the B2R upregulation by NGF, we tested the possible involvement of this receptor in NGF neuroprotective activity by analyzing CN survival (Figure 5). A quantitative analysis revealed that incubation of CNs with NGF (100 ng/mL) for 48 h (+NGF) did not alter cell viability, while 24 h of NGF deprivation (−NGF) caused a ~55% reduction in the number of surviving cells compared to control conditions (CTR). After adding back NGF (100 ng/mL) for 24 h, we observed a complete rescue of cell viability.

HOE140 (Icatibant), a selective B2R antagonist (1 µM), reversed the rescue induced by NGF replacement, demonstrating the crucial activity of B2R in NGF neuroprotection (Figure 5a).

Since it has been suggested that B2R is neuroprotective against different toxic insults both in vitro and in vivo [39,40,41,42], we treated CNs for 24 h with RPM-7 (Labradimil, Cereport), a specific B2R agonist (RPM-7 100 nM). As shown in Figure 5b, RPM-7 significantly rescued the cell death induced by NGF deprivation (−NGF), and this effect was antagonized by HOE140 co-administration (HOE140+RPM-7), confirming the neuroprotective role exerted by B2R in this experimental model.

Since we demonstrated an increase in the BK precursor gene Kng1 (Figure 1) in apoptotic conditions (−NGF), and the B2R antagonist was able to significantly reverse apoptosis due to NGF deprivation (Figure 5), we can hypothesize that in such conditions, endogenous BK could be produced by the cleavage of kininogen derived from Kng1.

To this end, we performed an ELISA analysis of cell media from CNs in control conditions (CTR), treated with NGF (+NGF), and deprived of NGF (−NGF). The results from the ELISA test indicated no differences among the groups (Supplementary Figure S1).

### 3.5. Electrophysiology

**Figure 6:**
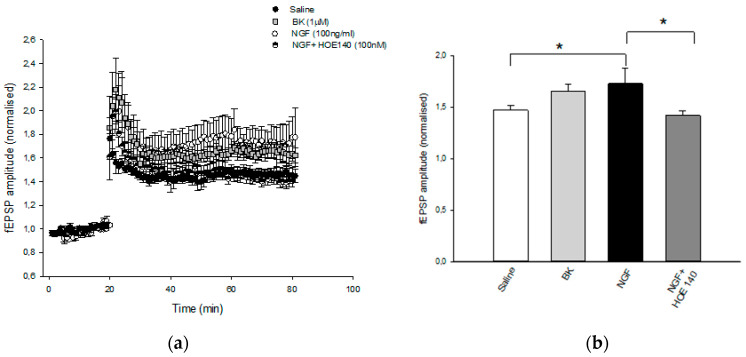
Effect of NGF LTP at CA1 hippocampal synapses. (**a**) Superimposed pooled data showing the normalized changes in field potential amplitude (±SEM) induced by HFS (100 Hz, 1 s). LTP is enhanced following BK (1μm) and NGF (100 ng/mL) treatment, and the latter effect is reversed by HOE140 (100 nM) application. At least eight slices from four mice were used for each group tested. (**b**) Histograms show the relative increase in the fEPSP amplitude measured 50–60 min post-HFS under the different pharmacological conditions (* *p* < 0.05).

Electrophysiology recordings showed that pretreatment of hippocampal slices with NGF (100 ng/mL for 1 h) was able to significantly enhance LTP at CA1 hippocampal synapses (1.77 ± 0.18) compared to the control (1.45 ± 0.06) (Figure 6). Notably, the application of BK (1 µM for 1 h) mimicked the NGF-mediated facilitatory action on CA1-LTP (1.64 ± 0.06). The effect of NGF was prevented by the pretreatment of slices with the B2R antagonist HOE140 (100 nM) (1.4 ± 0.05), further suggesting that NGF modulates synaptic plasticity via interaction with B2R. These results indicate that B2R, endowed with neuroprotective activity, plays a role in the NGF mechanism of action.

### 3.6. NGF Treated AD Mice

**Figure 7:**
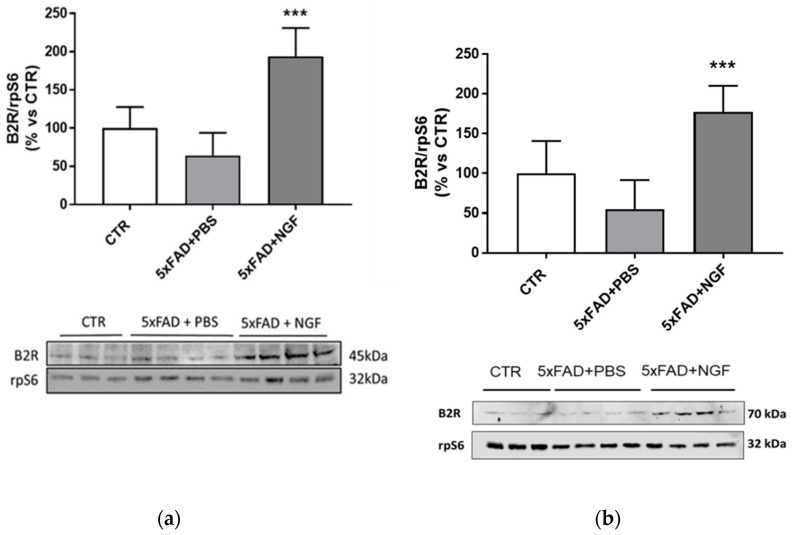
Overexpression of B2R by NGF treatment in 5xFAD brain extracts. Western blot analysis of B2R protein expression (panel (**a**) bands at 45 kDa; panel (**b**) bands at 70kDa) in brain extracts from wild type mice (CTR) and 5xFAD mice intranasally treated with PBS or NGF normalized against rpS6 and expressed as a percentage of the control (CTR). Data represent means (±S.E.M.) from four independent samples. Statistically significant differences were calculated by one-way analysis of variance (ANOVA) followed by Bonferroni’s test for multiple comparisons. (*** *p* < 0.001).

Considering the B2R upregulation by NGF treatment in vitro, we examined by Western blot analysis the effect of NGF chronically administered to 5xFAD mice. As shown in Figure 7, the B2R expression level in brain extracts was slightly detectable in the brain of wild type and 5xFAD mice treated with PBS, while its amount was significantly increased following NGF administration.

Both the 45 kDa band, corresponding to the nonglycosylated B2R, and the 75kDa band, consistent to the mature fully glycosylated B2R, showed about a four-fold increase, confirming the crucial role of this receptor also in vivo.

## 4. Discussion

NGF, discovered in the 1950s by Levi Montalcini and Hamburger, has been identified as a trophic factor for sympathetic and sensory neurons, inducing the survival, development, and neurite growth of these neurons [43]. It has also been reported that NGF deprivation of sympathetic neurons leads to their degeneration and massive death due to the activation of programmed cell death [44]. The NGF-promoting action found in sensory neurons was subsequently extended to a significantly and pronounced “trophic” effect in other nonsensory neurons [45].

NGF, synthesized in brain structures innervated by the basal forebrain cholinergic neurons and then retrogradely transported via axons to the bodies of cholinergic cortical neurons, is extremely important for their survival [46]. Indeed, the trophic support by NGF of developing and mature basal forebrain cholinergic neurons is required for normal plastic rearrangements during the development and functioning of adult cholinergic neurons, affecting the extent of connection between these and innervated targets [47,48].

NGF deprivation of these neurons leads to their degeneration, as demonstrated by evidence that the levels of NGF in brain structures are modified in neurodegenerative diseases, including AD [49]. The well-documented nociceptive activity of NGF, representing a substantial side effect for the development of an NGF-based prospective therapy for human diseases, is characterized by the upregulation of B2R expression in sensitive neurons [15,50].

Here, we demonstrated that also in nonsensitive cells (mixed cortical cultures), NGF was able to modify B2R expression. From microarray data, it emerged that following NGF exposure, B2R mRNA was significantly upregulated and its expression levels decreased in NGF-deprived conditions. In the same apoptotic conditions, B2R reduction was accompanied by an increase in BK and in inducible B1R mRNA levels, thus emphasizing that BK and B1R could be related to neurodegeneration, while B2R seems to have a neuroprotective role.

We established B2R upregulation by NGF also at a protein level; the results showed a selective expression in microglial cells. Since it is well known that B2R is widely distributed in rat brain within the neuronal compartment, including the cerebral cortex, our results could appear quite surprising. However, it is worth mentioning that while it was showed a specific B2R immunoreactivity in neurons in the whole rat brain, the expression of this receptor in glial cells could not be excluded [51]. In fact, the presence of B2R has been identified in rat primary cultures of cortical astrocytes [52,53,54] and microglia [55,56].

In the present study, we demonstrated that in mixed cortical cultures, B2R is expressed only in microglial cells, while no labeling was seen in neurons or astrocytes. On the other hand, by the use of the same mixed primary cortical cultures it was showed a weak expression of B2R immunoreactivity only in neurons [57]. Therefore, the observed discrepancies could be explained by different culture conditions, since in our experiments, we extended DIV and reduced B27 supplement concentration to amplify the effect of NGF.

Our results, indicating microglia as the main target of NGF activity, are in agreement with a previous work showing that following NGF treatment, microglial cells change into a neuroprotective and anti-inflammatory phenotype both in vitro and ex vivo [58]. Indeed, these authors demonstrated that NGF exerts its neuroprotective and anti-inflammatory effects on neurons, reducing cytokines levels, rescuing spine density and LTP deficit, in a microglia-dependent way, through the NGF receptors present in these cells both in vivo and in vitro [58].

The neuroprotective role of B2R was confirmed in this in vitro experimental model by examining the cell viability in apoptotic conditions obtained by NGF deprivation. We demonstrated that not only NGF, but also a specific B2R agonist (RPM-7), significantly rescued cell death. Moreover, HOE140 (Icatibant), a selective B2R antagonist, reversed the rescue induced by NGF replacement, indicating a crucial role of B2R in the neuroprotective activity of NGF.

Interestingly, HOE140 was able to significantly reverse NGF deprivation-induced apoptosis, suggesting the potential production of endogenous BK from kininogen cleavage, whose gene (Kng1) was demonstrated to be upregulated in this apoptotic condition. In contrast, ELISA analysis indicated no differences in the BK amount after NGF deprivation. However, we cannot exclude the possibility that BK could be effectively secreted, since it is well known that BK is rapidly metabolized by kininases including aminopeptidase P and carboxypeptidase N [59].

Moreover, Kng1 enzymatic processing can lead to the production not only of BK, but also of other metabolites such as des-Arg9-BK and Lys-des-Arg9-BK. These peptides are highly pro-inflammatory since they have greater affinity for the inducible B1R than the homolog pair BK, and could contribute to the neurodegenerative development seen in NGF deprived conditions [19].

Corroborating the crucial role of B2R in the NGF neuroprotective activity, electrophysiological experiments also indicated that the enhancement of LTP by NGF was significantly mediated by B2R, since BK mirrored the NGF action and the selective B2R antagonist HOE140 prevented the NGF effect. Since B2R is present in microglial cells, it reasonable to suppose that NGF exerts its effect on synaptic plasticity via microglia, in line with previous data showing that the ability of NGF to rescue chemical LTP is completely dependent on the presence of microglia in the cultures [58].

The involvement of B2R in the NGF neuroprotective activity was further demonstrated in vivo in four-month-old 5xFAD mice chronically treated with a NGF variant [38]. As recently proved by our research group, the administration of the variant neurotrophin by intranasal delivery, a noninvasive method to transport neurotrophic factors to the brain [60], was able to reduce neurodegeneration, Aβ deposition, and memory deficits, and to promote the rescue of synaptic plasticity in the 5xFAD AD model. Interestingly, the authors established that these NGF activities were mediated by glial cells, modulating inflammatory proteins such as the soluble TNFa receptor II and the chemokine CXCL12 [38].

In our experiments, we evidenced that in brain extract of 5xFAD mice, the mature, fully glycosylated B2R form was significantly upregulated by NGF treatment, indicating a key role of this constitutive receptor in the mechanism of action of NGF.

Considering the selective overexpression of B2R in in vitro microglia, the results obtained in brain of 5xFAD treated mice indicated the same localization also in vivo. Of note, as already demonstrated [38], both NGF receptors p75NTR and TrKA are upregulated on 5xFAD microglia, as well as on human AD microglia, supporting the role of these cells as a target for the NGF neuroprotective activity.

Additional experiments should be performed to characterize the molecular mechanism through which the increased B2R is involved in NGF-mediated neuroprotection in order to identify new potential therapeutic strategies for AD.

## Supplementary Materials

The following are available online at https://www.mdpi.com/2073-4409/9/12/2651/s1, Figure S1: Determination of BK levels in CNs media from cells in control conditions (CTR), treated with NGF (+NGF) and deprived of NGF (−NGF). Results, expressed in pg/mL, represent means (±S.E.M.) from 4 different experiments run in duplicate.
