# Supplementary material for: Involvement of Bradykinin Receptor 2 in Nerve Growth Factor Neuroprotective Activity
Source: Cells. 2020 Dec 10;9(12):2651. doi: 10.3390/cells9122651 (PMC7763563; doi:10.3390/cells9122651)
Supplement: Supplementary file 1 [file cells-09-02651-s001.pdf]

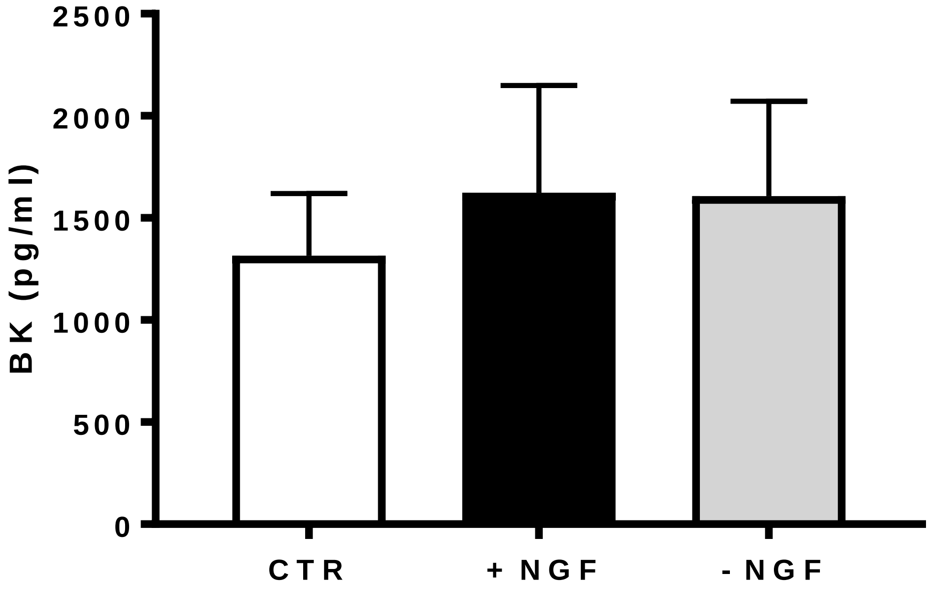

Supplementary Figure 1. Determination of BK levels in CNs media
from cells in control conditions (CTR), treated with NGF (+NGF) and deprived of NGF (-NGF). Results ,expressed in pg/ml, represent means (±S.E.M.) from 4 different experiments run in duplicate.
